# ‘I think my body has become addicted to those tablets’. Chronic heart failure patients’ understanding of and beliefs about their illness and its treatment: A qualitative longitudinal study from Uganda

**DOI:** 10.1371/journal.pone.0182876

**Published:** 2017-09-28

**Authors:** Elizabeth Namukwaya, Scott A. Murray, Julia Downing, Mhoira Leng, Liz Grant

**Affiliations:** 1 Department of Medicine, Makerere University College of Health Sciences, Kampala, Uganda; 2 Primary Palliative Care Research Group, The Usher Institute of Population Health Sciences & Informatics, The University of Edinburgh, Medical School, Edinburgh, United Kingdom; 3 Global Health Academy, The Usher Institute of Population Health Sciences & Informatics, The University of Edinburgh, Medical School, Edinburgh, United Kingdom; Universite de Bretagne Occidentale, FRANCE

## Abstract

**Background:**

Patients with heart failure in Uganda present for health care with advanced structural heart disease, have repeated hospitalizations and poorly controlled disease symptoms. The reasons for these are unclear. Literature from other settings shows that patients’ understanding of their illness and their beliefs influence their health related behaviour. The study aimed to explore the beliefs of patients with heart failure, their understanding of their illness and its treatment, and how this influenced their health related behaviour to inform future health education programs, information and palliative care services.

**Methods:**

Serial qualitative in-depth interviews were conducted with Heart Failure patients who were purposively sampled and recruited in Mulago National Referral Hospital until thematic saturation was reached. In-depth interviews were conducted at three time points over the course of their illness with intervals of 3 months between interviews. A grounded theory approach was used in data analysis. The University of Edinburgh ethics committee, Mulago Hospital Research Ethics committee and the Uganda National Council of Science and Technology (Reference numbers D/GC/178; MREC 33, SS 3083 respectively) approved the research.

**Results:**

A total of 40 face to face qualitative longitudinal interviews (36-patient alone, 4 paired-patient and family carer), were conducted with 21 patients. The findings revealed that heart failure patients were unaware of the symptoms of the illness and their definition of illness differed from that of health professionals. Patients understood their diagnosis, cause of illness, prognosis and the importance of the medicines differently from health professionals, and had insufficient information on self-care. Lay beliefs were used to explain many aspects of the illness and treatments. All these influenced where patients sought care and their adherence to treatment, self-care and follow up leading to uncontrolled disease.

**Conclusion:**

There is a high level of health illiteracy among heart failure patients in Uganda. Patients rely on lay beliefs to make health decisions and medical information is often miscomprehended. There is an urgent need for health education using culturally appropriate information.

## Introduction

Cardiovascular diseases (CVDs) contribute to most of the mortality attributed to Non Communicable Diseases (NCDs) globally. Among CVDs, heart diseases are the leading cause of mortality globally, accounting for 66% of CVD deaths in males and 62% of CVD deaths in females. [1] Patients with heart disease in Africa commonly present with Heart Failure (HF), [2] in the advanced stage requiring hospitalisation [3, 4]. The course of illness for HF patients in Africa is dominated with repeated hospitalisations [5] due to poorly controlled end-stage disease. The reasons for delayed presentation for health care and repeated hospitalisations are unclear. However, literature from other settings shows that patients’ understanding of their illness and the beliefs they hold about their illness influences their health related behaviour as people draw on these beliefs and knowledge to make sense of their symptoms. [6–8] Patients’ understanding of their treatments is also a predictor of their adherence to treatment and self-care and this affects their outcomes. [7] Illness perceptions also influence patients’ psychological response to the illness and these perceptions have been found to be associated with psychological well-being in HF patients outside Africa.[9] There are no published data in the Ugandan setting that describe HF patients’ understanding of and beliefs about their illness and treatment. This study therefore aimed to explore Ugandan HF patients’ understanding of their illness and its treatment and how this influenced their health related behaviour. The findings from this study will inform future health education programmes and patient information services and will provide information for palliative care providers who engage in complex communication with these patients. This study addresses a gap identified by Selman et al in a review of literature on HF in Africa in 2015 which highlighted the need for culturally sensitive research on patients’ experiences to explore if issues such as communication difficulties observed in high-income countries also exist for HF patients in Uganda.[10]

## Methodology

### Setting

The study was conducted on the general cardiology ward in Mulago National Referral Hospital (MNRH) in Kampala, Uganda, and in patients’ homesteads from March 2013 to January 2014. Patients were recruited in hospital and all first interviews were conducted there. Subsequent interviews were conducted either at the patient’s home or in hospital.

### Theoretical framework

In order to generate interventions that are context specific we adopted the epistemological stance of constructivism.[11,12] Constructivism asserts that people develop subjective meanings of their experiences and therefore meanings of experiences are multiple and are influenced by their social, cultural and historical context. [11,12] Our assumption was that people’s understanding of and beliefs about their illness are subjective and multiple. An interpretivist theoretical perspective was adopted because it resonates well with constructivism as it enables eliciting rich information related to individual perceptions of issues. [13] The principles of the constructivist grounded theory methodology were employed in sampling of participants and data analysis. This methodology generates analytical interpretations of data and is in line with the interpretive theoretical perspective.[14] The constructivist grounded theory approach was chosen because it keeps the participants voice and meaning in the final research outcome.[15] This resonates well with the principles of palliative care that aims at giving voice to the participant.

### Methods

Serial qualitative in-depth interviews were conducted with HF patients so as to get an in-depth insight into patients’ beliefs and understanding of their illness and how these change over their illness course.[16,17]

### Sampling procedure

Patients were purposively selected in order to represent the local demography of New York Heart Association stage 3 and 4 HF. Patients were eligible if they were 18 years or older, had at least 4 out of the 5 criteria of the definition of advanced HF by Metra et al [18] and lived within 30 km from Kampala to enable follow up. Patients were excluded if they had diminished cognitive capacity to consent, if they were too ill to be interviewed, had profound communication deficit or if they could not speak Luganda or English, which are the languages spoken by the researcher.

### Data collection procedure

Participants were interviewed during the time of hospitalisation, and were followed up monthly by mobile phone to maintain contact and relationship and to provide an alert if there was a change in health status. Change in health status (as judged by the researcher and the participant) would trigger a subsequent interview in order to capture evolving understanding and beliefs when symptoms changed. Repeat interviews were conducted at 3 and 6 months thereafter if the patients’ clinical condition remained stable, and earlier if there was marked deterioration. All interviews were audio recorded and field notes were written. The interview guide used for the study is attached as S1 Appendix. This interview guide was used for a larger study that explored HF patients’ experiences over their illness course which had one of its objectives as understanding patients’ beliefs about their illness and its treatment.

### Analysis

Participant interviews were first transcribed to Luganda and then translated into English by a secretary in the palliative care unit who is fluent in both languages. The first author who is also fluent in both languages read through all the transcripts and re-listened to the audio interviews to confirm completeness of transcription and accuracy of translation. Transcripts were exported into QSR Nvivo software version 10 together with the field notes. Analysis started on completion of the first interview so that emergent themes could be incorporated into subsequent interviews, until data saturation was achieved. Principles from Charmaz’s grounded theory [19] were employed as follows; line by line coding was done to identify initial codes that corresponded to themes that described the data, this was followed by focused coding where larger segments of data were condensed to identify categories and in so doing the initial codes were moved from a descriptive to a more conceptual level. Constant comparison was employed throughout the coding process in order to identify similarities and differences by comparing data with data, and incident with incident of a participant, within the same interview and in different interviews of the same participant over time. Comparison was also done with all participants across the group [19] which helped in identifying patterns and contrasts. Finally theoretical coding was done where each of the codes generated in the focused coding were examined to determine how they related to each other as hypotheses to be integrated into a theory. [20] The findings of this paper are part of a larger study that explored HF patients’ experiences over the course of their illness and a theory was generated for the overall study but not for these findings.

Four authors discussed and agreed on codes and themes that were generated from the data. QSR Nvivo software enabled organising and working on large volumes of qualitative data. The approach to analysis of qualitative longitudinal interviews proposed by Thomson and Holland [21] as well as that proposed by Saldana were employed as described below.[22] Analysis was done at the end of each interview for each participant. On completion of all the participant interviews in a given serie,(e.g completion of all first interviews for all participants) a summary of the analysis was written for that group of interviews and main themes were identified. Cross-sectional analysis was done across all interviews for each series of interviews (that is first, second and third interviews) to capture each moment in time [21] At the end of the longitudinal interviews all the analyses for each of the serial interviews for each participant were drawn together to form a ‘case profile’ so as to compare themes across time for an individual but also for the group.

### Ethical approval and consent to participate

Approval for this study was provided by the University of Edinburgh ethics committee, Mulago Hospital Research Ethics committee and the Uganda National Council of Science and Technology (Reference numbers D/GC/178; MREC 33, SS 3083 respectively). Permission to access participants was obtained from the administration of MNRH and the ward in-charge of the cardiology ward. The participants provided written informed consent to participate in the study by signing or applying a thumb print. They provided written consent for anonymised transcribed data from their audio-records to be published.

## Findings

**Fig 1:**
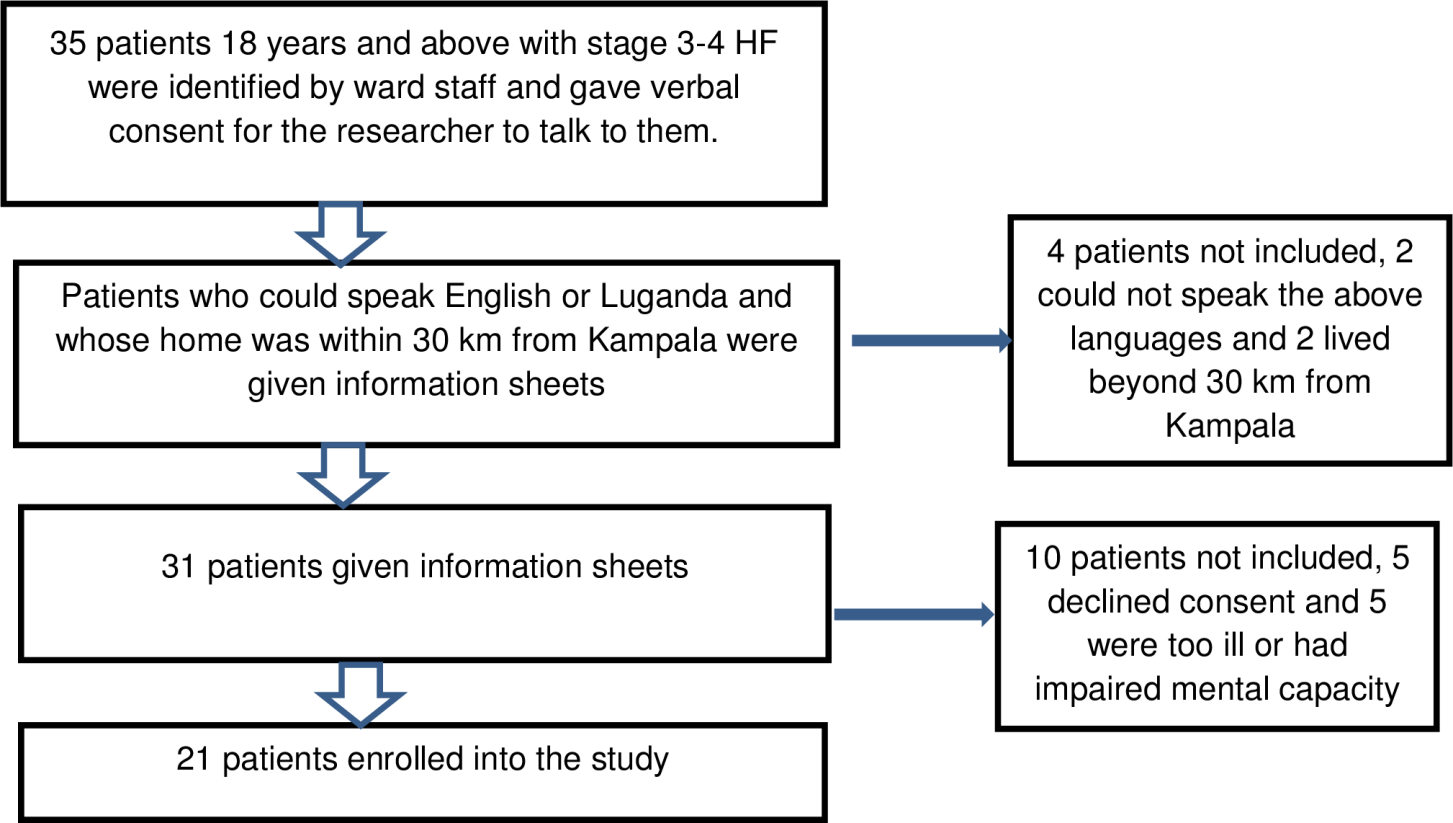
Flow chart of patient recruitment.

Thirty-five patients were approached who met the inclusion criteria. Two patients could not speak either of the two languages in which the interviews would be undertaken (English or Luganda) and two patients homes were out of the 30 kilometres radius span from Kampala, thus follow-up interviews would not be feasible, and therefore could not be included in the study. Information sheets were given to thirty-one participants. Five patients declined to consent to the study, three reported having been involved in another research before that and were not ready to answer more questions, and two did not give any reasons for declining to consent. Five patients were excluded because they were too ill or were too breathless to talk, or had impaired mental capacity and were not able to comprehend the information sheet. Twenty-one patients consented and were enroled for the study. The patient recruitment is summarised in Fig 1.

A total of 40 face to face qualitative longitudinal interviews (36-patient alone, 4 paired-patient and family carer), were conducted with 21 participants. All interviews were conducted in Luganda. The socio-demographic characteristics of the participants are summarised in Table 1 below.

**Table 1:** Characteristics of those who participated in the study.

Characteristic	N = 21
**Age group**	
18–20	1(4.8%)
21–30	7 (33.3%)
31–40	3 (14.3%)
41–50	3 (14.3%)
51–60	6 (28.5%)
61–70	1 (4.8%)
**Sex**	
Female	15 (71.4%)
Male	6 (28.6%)
**Education level**	
None	5 (23.8%)
Primary	8 (38.1%)
Secondary	7 (33.3%)
Tertiary	1 (4.8%)
**Marital Status**	
Single	8 (38.1%)
Married	6 (28.5%)
Widowed or separated	7 (33.3%)
**Diagnosis**	
Dilated cardiomyopathy	4 (19.1%)
Endomyocardial fibrosis	2 (9.5%)
Hypertensive heart disease	6 (28.5%)
Rheumatic heart disease	7 (33.3%)
HIV Cardiomyopathy	2 (9.5%)

The timing of the face-to-face and telephone interviews and patient deaths is shown in S2 Appendix.

Two main themes emerged underpinning participants’ understanding and beliefs of their illness that is limited health literacy and lay knowledge. These two themes were very interlinked. The definition of Health literacy developed by the European health literacy consortium in 2012 was adopted for this study. Health literacy was defined as “people’s knowledge of health information, their motivation, and competences to access, understand, appraise and apply the health information in order to make judgements and take decisions in everyday life concerning health care, disease prevention and health promotion to maintain or improve quality of life during their life course.”[23] The subthemes under the theme of limited health literacy included: lack of knowledge and competency to interpret symptoms as due to ill health,.limited competency in appraising and comprehending of given health information and lack of information on the purpose and importance of the different medicines.

Lay knowledge was defined as “the ideas and perspectives employed by people to interpret their experiences of health and illness in everyday life” a definition proposed by Gareth Williams. [24]

Subthemes under the theme of lay knowledge included; lay definitions of disease and health, the traditional and complementary medicine paradigm, influence of spiritual, religious and cultural beliefs and lay perceptions on self-care.

### Limited health literacy

Participants’ interviews revealed that they had limited health literacy particularly regarding recognition of the symptoms of heart disease and the purpose of their treatments. They struggled to comprehend the information that was given to them by health providers.

#### Lack of knowledge and competency to attribute initial symptoms to ill health

Many participants did not have knowledge about the initial symptoms of HF and did not associate the symptoms they were having to any form of illness. They therefore did not seek early medical care, instead interpreting symptoms as due to old age, coexisting conditions such as HIV and its treatment and pregnancy

“I *really did not know much about this illness and so when it started I did not really take much notice” (Patient 20, Interview 1)*

*“I thought maybe this menstruation coming to an end is associated with some problems of the heart ….we are told you get problems, you get a lot of heat, you sweat and all those symptoms I had them.* A*s you grow older some things change in your body, you cannot be the same as before when you are young so I thought that was the problem.*

‘I also thought it may be the HIV medicines I was taking.’(Patient 6, Interview 1).

‘During the eighth month of pregnancy I felt so heavy. I could not even lift a pot of food and put it on the fire, everything I did my heart would beat so much. There were some women, the traditional birth attendants I went to one of them and explained my situation and she said, no it is okay the child has a normal lie in the abdomen but might be big or is seated in a lot of water. So I thought it was the pregnancy.’ (Patient 5, Interview 1)

The intermittent nature of symptoms, an overall sense of healthiness when not engaged in manual work, and initial presentation with symptoms such as cough which were regarded as minor and did not cause concern further compounded this difficulty in recognition of the illness.

“I did not understand, initially I thought it is something that has come and will go because it would come and if I stood and rested it would then go away then I would start walking again, just like that”. (Patient 8, Interview 1)

“When it started I thought it was the mere cough which came on its own” (Patient 4, Interview 1)

Often a number of factors existed simultaneously, making self-assessment complex.

#### Limited competency in appraising and understanding of information given on the diagnosis, cause and treatment of HF

Participants described pondering on their diagnosis, the cause of the illness, their treatment and prognosis and they interpreted these in their local context. Their understanding evolved over the course of the illness and was influenced by the information they received from lay people and health professionals, their past and present experiences, their previous knowledge and their level of health literacy.

A major finding was the different understanding participants had of their illness compared to the information health professionals had recorded in their ward notes. Participants’ narratives of their understanding of their diagnosis integrated sporadic medical knowledge which was frequently misconstrued. For example a participant described what appears to be rheumatic fever with an in-depth description about how her “blood was not flowing”, another described her illness as having “cracks on her heart “.

‘I was then told the heart was tired and they told me that it was fever of the joints that led to the heart disease and the heart vessels are abnormal they were scarred and so blood does not flow.’ (Patient 19 interview 1 -had rheumatic valve disease)

‘When I asked the person who was examining using the TV (ultra sound scan) of the heart on me what he had found he said we have found cracks and some of blood leaking on my heart, and he said if you take your medications they will go away.’ (Patient 11 interview 1 –patient with hypertensive cardiomyopathy)

Most participants reported that they their heart was “surrounded by water”. While pericardial effusion could be described as such in lay language, no participant had pericardial effusion. Rather this term appears to reflect how lay people in this context conceptualise HF.

‘My daughter took me to hospital ………… they found I had water in my abdomen and they sucked it out from this side, (points to the right). From then I understood that my heart was surrounded by water because the health care worker explained to me I had water in the abdomen.’ (Patient 10 interview 1—had no pericardial effusion)

The similarities in presenting symptomatology of those with pericardial effusion and HF could explain how this lay diagnosis had become so popular. Participants feeling bloated and seeing the body swelling, with background knowledge from their health care professionals that they had heart disease and water elsewhere, made assumptions that the origin of the water was around their heart.

While participants listened to the information they were given by health professionals their limited competency in appraising and understanding the information meant that they frequently re-interpreted parts. Having insufficient information from the health professionals and the difficulty of translating and communicating the concept of HF to participants, especially the challenge of finding the appropriate words to use for HF and other heart diseases in the local languages contributed to the misconstrued understanding.

Participants tended to relate the cause of their illness to events or circumstances in their past lives but did not refer to medical knowledge on the aetiology of heart disease. Some participants’ explanations of the aetiology showed having traces of medical information. Only one participant’s understanding of the aetiology of his HF was concordant with the notes in his file. This participant had a higher level of education than the others and appeared to have been more motivated to seek information on his illness. HF was attributed to:

A diet with a lot of salt;

‘The person on radio said if you add raw salt to food this will cause water to accumulate around your heart. Looking back when they said this I realized I ate a lot of raw salt long ago, when I was pregnant I would eat crystals of raw salt … it was my fault.’ (Patient 1, Interview 1)

Doing manual work without rest;

‘I think working too much, I used to work a lot I grew up in the village and therefore I had to dig, such things, may be being in the sun for the whole day was one of those things which caused it. I am not sure really.’

(Patient 14, Interview 1)

Diseases such as hypertension and HIV;

‘The pressure went up so high and hit my heart and my kidneys and destroyed them, the lungs are destroyed because the heart is not working well the liver is also in trouble.' (Patient 20, Interview 1)

‘But in my observation I think in all this is HIV. I think it is HIV that is causing the suffering; I may want to get an excuse that it is the heart but I think it is HIV… I think that HIV manifested as heart disease because sometimes it hides somewhere. Even the doctors tell me the heart is not really bad ….on the medical form they always write HIV. They often ask me where I get HIV care to make sure I am getting treatment for HIV.’ (Patient 6, Interview 3)

A complication of childbirth as the baby moved through the womb;

'The illness started with delivery of the child I have told you, I had problems with delivery,…. when the baby was turning in my womb he kicked my heart, I felt bad during the delivery I felt chest heaviness in labour and told the midwife to come and check because I felt the child had pushed my heart but she ignored me and she just continued sleeping…. When I was hospitalized sometime after the doctor asked me if someone had ever hit me on the heart and I said no..but then I remembered what happened during delivery …the baby had kicked me on the heart.’ (Patient 17, Interview 1)

Worrying a lot and a past history of being battered as a child;

‘In my view, I think what caused it was too much worry in the past. I remember I had lots of problems and now this was on top of the others I felt that my heart was heavy with sadness, so I think looking back that this might have been the cause.’ (Patient 5, Interview 1)

‘I even thought to myself this illness came because I was beaten a lot as child, I might have been beaten in a bad spot.’ (Patient 12, Interview 1)

Many participants thought they were going to be cured.

‘As usual I expect them to cure the problem.’ (Patient 8, Interview 1)

Others knew it was a chronic illness but expected a normal life span.

‘I expect to improve, not to get cured,…..this illness has come in my old age, so this will be with me until I die, because there is no illness you get in your old age and expect that it will go away.’ (Patient 6 interview 1, 50 year old with illness for 3 years)

Participants’ understanding of their prognosis changed with time probably because of their experience of living with the illness, observing peers, but also their continued interface with HPs could have led to getting more information leading to changed expectations as illustrated in the example of participant 3 who started by hoping that he was going to get cured but later on, along the disease trajectory, started referring to death implicitly.

‘Most of the time I hope for cure not just improvement.’ (Patient 3 interview 1)

‘I am worried but I am just grateful that all my children are grown. The last one is in senior 4 at least he can stand on his own if anything happens.’ (Patient 3, Interview 2)

Participants had different perceptions and beliefs of the medical treatment they received and this influenced their adherence to it. The experiences reflected lack of knowledge of the purpose and importance of the different medicines.

Most participants believed medications that reduce symptoms, such as swelling were the most useful ones and therefore over time they selectively chose to take those they thought to be useful especially if they had to buy the medications and did not have sufficient funds.

‘If I have those water tablets, you can deny me any other tablets but give me those tablets because they give a lot of relief’ (Patient 1, Interview 1)

This was problematic when participants were on multiple medications and were guessing which ones improved symptoms, and sometimes they chose the wrong ones.

‘These are the ones which help (points to ciprofloxacin) they help me pass a lot of urine, those (furosemide) were given to me but did not do anything…;yes the 10, the last time he came he was given 90 of the other and 10 of these (ciprofloxacin) but the former run out without making any difference, but the 10 made a difference and I even went back to buy more of them (ciprofloxacin) when they run out’ (Patient 10, Interview 1)

In this example the participant believes that a short course of prescribed antibiotics was of more value in reducing his body swelling than the diuretic he was on, thus stopped the latter, but continued to independently buy and take the former.

Participants also believed that injections were superior over oral medications

‘But the water tablets are weak, …you swallow then but you do not pass out the water you continue swelling, you need an injection.’ (Patient 6, Interview 1)

After taking diuretics for a long time some participants believed they had stopped working on them or that they had developed tolerance to them and this reduced their motivation to adhere to them.

‘I think my body has become addicted to those tablets and they do not work on me any more so I had to stop them. They first gave me first 40 mg then they increased to 80mg but then I was still failing to pass urine. I got worse so I stopped them’ (Patient 20, Interview 2)

Some participants believed that some treatments were associated with imminent death such as oxygen and that invasive treatments such as paracentesis caused more fluid to accumulate in the abdomen and this would set off a vicious cycle of fluid accumulation and frequent paracentesis and therefore refused such treatments or if they had them, they became anxious.

‘One day they wanted to take me to have oxygen and I thought… yiiii. How will they put my heart on that machine and I still continue to be alive….. to be sincere I was scared of it.’ (Patient 9, Interview 1)

‘Also as you know they removed water from my abdomen and I was told that if they start sucking out the water in your abdomen then it will continue coming back all the time and you have to come back here all the time which worried me.’ (Patient 5, Interview 1)

### Lay knowledge

HF and its symptoms were conceptualised by participants, and the community in various ways. Some of these lay conceptions drew from a repertoire of prevailing lay beliefs and knowledge, lay experiences of those who had similar symptoms and from religious beliefs.

#### Lay definition of illness

The lay definition of illness differed from that of health professionals. Illness was defined not only by the patient but by their families and communities who collectively agreed the definition of illness. Having symptoms that were not serious enough to prevent a patient from working was not seen as an indicator of illness. Many participants lived with a range of symptoms for 3 months before approaching a health professional. Illness was defined by having a significant symptom experience which included;

Symptoms which significantly impaired functioning and activities of daily living;

‘I used to have pain in the legs and the back, I thought it may be kidney disease or something but I just thought about it but did not make any effort to do what …to go to hospital, I just went on with my duties as usual until I felt worse,….. I felt chest pain, I could not carry things I used to carry I used to get something like difficulty in breathing …. I could no longer work, then I decided to go to hospital” (Patient 3, Interview 1)

Symptoms which were legitimized and affirmed by families, friends and community neighbours. Usually signs of illness such as abdominal or leg swelling were needed to convince others that one was ill.

"During the first 3 months I was very unwell at night but people would see me looking okay during the day and thought I was well. I told my friends and neighbours that I could not sleep at night and when I tried to lie down I felt very bad. ……it was not until after 3 months, when my abdomen started swelling….. when I showed it to my son then that night I was taken to hospital” (Patient 1 Interview 1)

Developing symptoms reminiscent of the symptoms of diseases that the community feared, for example tuberculosis;

“My friends started telling me that I might have tuberculosis and took me to a hospital” (Patient 5, Interview 1).

Symptoms which developed acutely and severely, such as those associated with death, for example breathlessness;

“That *is why I went to hospital because as a human when you are breathless you know death is near” (Patient 16, Interview 1)*

Symptoms not amenable to local remedies

“I have had the problem for long but tried to push on, you know for us in the village we start with using local herbs, you are told take this but then I went on and started having palpitations and sweating, I got local herbs to help with the palpitations and they decreased then they started all over again. Then I felt my abdomen was swelling and then I went to a clinic” (Patient 10, Interview 1)

Once a person was defined as being ill and unable to work, care was sought. Therefore in most cases this was delayed because visible symptoms and gross impairment of function only came with advanced disease.

#### The traditional and complementary medicine paradigm

Most participants initially drew on the traditional and complementary medicine paradigm to conceptualise the changes in their health and therefore they started off by using herbs when they had symptoms. They only sought medical care when symptoms became worse as illustrated in the quote below.

“I have had the problem for long but tried to push on, you know for us in the village we start with using local herbs, you are told take this but then I went on and started having palpitations and sweating, I got local herbs to help with the palpitations and they decreased then they started all over again. Then I felt my abdomen was swelling and then I went to a clinic” (Patient 10, Interview 1)

Participants commonly used alternative medications alongside biomedical treatments, though this information was not initially volunteered. This may be because they sensed a tension between the different forms of care and the healthcare givers in Uganda. One participant withheld this information completely, until it was disclosed by their family carer at the end of his first interview. Herbs and traditional treatments were used for different purposes:

To take away evil curses or spells;

‘I took some herbs but not from a witch doctor but I got them from a herbalist, who told me that I had got or been bewitched with a condition called ‘entumbi’ (associated with abdominal swelling).’ (Patient 13, Interview 1)

To enhance the effects of the conventional medicine or to mitigate side-effects of the conventional medicines.

‘I was told to buy the herb called the heart of the soil by a herbalist who cooked it for me and added some other herbs in it and told me to drink it. But now I see some improvement in the heart….yes, it is a good treatment for the heart, have you heard of the heart of the soil? If the strength of the injection has gone down then you add in this herb but then you realise that when you take them together it removes all hidden illness and you get diarrhoea.You feel a bit better and then you can continue with the hospital medicine it is like mixing some meat with a bit of fat when you are eating.’ (Patient 6, Interview 3)

‘I have been taking some herbs I made myself to give me some energy, I have them here and I was told to take them to help me deal with the strong medicines, that will be given to me in the hospital.’ (Patient 11, Interview 1)

And as an alternative to the conventional medicines.

‘If I have not swallowed the medicines at night I swallow garlic, do you know it? I cut it into small pieces and swallow it like tablets, there I can sleep ….. It lowers the blood pressure as long as you eat enough food to make you full, you can swallow the garlic every day it has no problem. Dr.Sp the herbalist do you know him? He told us that if you swallow hot pepper it helps, as long as you take it and follow it with a drink of water.’ (Patient 11, Interview 1)

Participants often started with using herbal treatments, but lost faith in them when their illness did not improve. Others used alternative medicine when they felt the conventional treatments were failing and this was often at the advice of other people.

‘They gave me the that native medicine when the illness had just started but aah it did not help’ (Patient 18, Interview 1)

Participants’ choice to use herbs and the reasons for their use such as mitigating side-effects of conventional medicines and using them as alternatives to medical treatment led to poor adherence to conventional treatment.

#### The role of faith, cultural and religious beliefs

People’s faith, religious and cultural beliefs also influenced their perceptions of the cause of the illness and its treatment.

Some participants perceived illness as a test of their faith in God believing what was happening had been predestined by God. Others believed Satan as the cause of their illness and suffering.

‘Nothing comes without His (Gods) knowledge, He knows, sometimes He brings it (illness) as a test, sometimes He tests us to see what your ‘heart’ is like.’ (Patient 3, Interview 1)

‘For me I think Satan brings (the illness) and God removes. Satan has to treat us as he did to Job. Do you think the Lord made Job sick?(patient 1 interview 1)

Those who also believed in traditional religions related the cause of illness to witch craft and evil charms, this was observed when participants had particular symptoms such as swelling of the body which is attributed to witchcraft by some cultures in Uganda. This led to some participants choosing their initial care from witch doctors.

‘As you know in the village people thought I had been bewitched, I even tried and went to a witch doctor…. After a while my family realised it was not getting better and so they brought me to Mulago hospital” (Patient 13, Interview 1)

‘My aunt, she thought I had been bewitched.’ (Patient 2 interview 1)

Some participants stopped taking their medications because they believed that taking medication was a sign of a lack of faith in God, and faith was very essential for one to be cured.

‘The pastors have been praying for me but most problems have changed now. Do you know that I do not take my medicines these days but my blood pressure is normal it surprises me too.…… It is because my pastor prayed for me, he gave me a prayer and asked me to believe so I follow, yes, but well it mostly depends on your faith, if they pray for you and you believe that you are cured.’ (Patient 20, Interview 1)

#### Lay perceptions of self-care

Many participants took extra measures in addition to the medical treatment given to ensure they improved their well-being; some felt they were in a battle with the illness and self-care was their artillery. Participants’ perceptions of self- care reflected the contemporary lay views on what good health is and how to achieve it, but were not specific to HF. They understood good self–care to include the following;

Having what they considered as an appropriate diet. They considered processed and packed foods superior to local foods such as yams that were considered bad food.

‘I heard over the radio that if you add raw salt to food this will cause water to accumulate around your heart so I avoid it. If I am at home I drink a lot, I take ribena, lucozade, and I can access tomatoes and care for myself. I use a lot of packed drinks. I try to eat well; sometimes the food you eat may not be good for a sick patient for example yams.’ (Patient 1, Interview 1)

Avoiding what they perceived as precipitating factors such as worry and alcohol.

‘One should avoid things which scare or worry him, even when he is bereaved do not give the news badly because it may frighten them alot, so bring the news gently. Also these children who play and shout or make noise they frighten you and then you get palpitations. The food is difficult because one becomes very choosy, you may want something but by the time they bring it you no longer want it. This happens to me a lot.’ (Patient 10, Interview 1)

Having time to rest, adhering to treatments, having regular reviews in hospital and being physically active. The motivation for self-care was to avoid being dependent, and some participants felt more responsible than others if they cared for themselves.

‘But one important thing is that I wake up early and get my broom and sweep so that I can breathe in the fresh morning air and then I cook my food which I eat and then I can take my medication. Then I go on to rest a bit have a nap, then when I wake up I go and pick coffee berries in the garden, so I try to be physically active so that I do not become like porridge or to be ill as a prince or princess. Who would carry me; you would be bothering/ burdening your close people. That is it. I also walk around in the village, do evening walks. Now with the heart, they tell us to eat fruits, we should not eat salt, no water; this illness is so selective. I have been fighting my war. If you do not care for your life you are gone with this illness.’ (Patient 6 interview 1)

Participants felt medical information sometimes conflicted with what they understood as healthy living for example most health messages emphasise having enough fluid in the body to be healthy and to get strength and yet they were being told to limit their fluid intake as illustrated in the quote below.

‘They tell us to drink less but remember the other medicine that they give us dries the throat you have to drink when you take it, so it is a bit difficult to balance the two, you have to be careful. Because at my age I cannot eat a lot I drink more. So I have to drink what will help me gain strength? When I eat in the morning then I do not eat again. Therefore I have to drink, after all even when we eat solid food it turns into fluid in the body even fluid remains fluid in the body so it is the same really eating or drinking. Most of our body is made of water. It plays a big role in our body.’ (Patient 6, Interview 3)

## Discussion

People with HF in Uganda had different understandings and beliefs about their illness and its treatment than health professionals. This influenced their health seeking behaviour and adherence to treatment. Having symptoms did not equate to having an illness. It was only when symptoms severely affected functionality, or if they were seen and legitimised by their community, or were associated with an imminent danger of death or were potentially infectious to others, that they were seen as pointers of illness. These findings are consistent with research in India among the Bondos tribe which [25] found that people defined illness as being unable to work, feeling weak and being non-functional, and health as being able to carry out social roles. The social-economic conditions of these people were similar to that of those in this study’s population and may explain the similar findings. In Uganda and in India, where survival is dependent on work, illness may need to be “put off” until one is non-functional because of the need to continue to work. HF symptoms among our participants were sometimes attributed to old age in line with a study in German where the elderly did not regard HF as genuine disease but as due to old age. [26] Our findings of the illness definition being determined communally are in accord with the findings of Uskul and Ayse. [27] The findings of patients and health professionals having different definitions of illness also fit into Kleinman et al’s illness explanatory model [28] that suggests that labelling of one’s changed body feelings (symptoms) by one’s self or one’s family as illness is important in defining illness, and this will depend on the patient, family and community’s perceptions and evaluation of these bodily changes and how they explain illness. Kleinman et al argue that disease (*‘abnormal psychophysiological function’*) should be differentiated from illness (*‘personal*, *interpersonal and cultural reactions to discomfort or disease’*) as illness is a cultural construct [28]. They go on to explain that health professionals view the disease as the problem but patients are concerned with the difficulties in living due to the sickness. Bury explains that people test the meaning attached to their bodily changes with their experiences with others first because they are not sure if they will have shared perceptions of the same situation as others, and therefore illness is a communal not a personal construct.[29] Bury, Uskul and Hynie and Kleinman, Eisenberg and Good’s theories, therefore, explain delays in defining and recognizing illness in our study because of the need for legitimacy and cultural definitions of illness. These are significant messages for health educators and health professionals to understand because participants’ definition of illness impacts when care is sought and will determine if one continues with their medical treatment or not.

A major finding was the limited health literacy and major reliance on lay knowledge to explain health related matters. This is consistent with research among ethnic minorities with HF in the United Kingdom who were found to have limited healthy literacy particularly limited understanding of their illness. [30] There is a paucity of data on health literacy in Africa, but the available data is consistent with low health literacy. [31] This should be a major public health concern as low health literacy among HF patients has been found to be a barrier to self-care and is associated with increased mortality [32].

Health illiteracy was a major contributor to the late recognition of illness, late seeking of medical care and a determiner of where care was sought. Limited understanding of the illness led to assuming non-medical causes of the illness and delay in seeking medical care. Health illiteracy also was a factor in the improper use and sometimes abandonment and the refusal of medicines for treatment of HF, misinterpretation of health information given and inappropriate self-care. This finding of health illiteracy being related to poor treatment adherence and poor self-care is widespread. [33,34] The findings of participants’ failure to comprehend medical information have important implications on how health professionals should conduct health education and provide information to people. Information should be appropriated to people’s level of education and should take into account what they know, their beliefs and the knowledge they have acquired from their lived experiences with the illness, this will ensure common ground between patients and health professionals and better acceptability of information from health professionals. As we observed in this study, people drew on lay beliefs and knowledge to make health decisions. For example, cultural beliefs were used by many participants to; explain their symptoms, define illness (as explained above) explain the illness cause, diagnosis and prognosis, determine which medicines were appropriate for use and determine care for self. Cultural and lay beliefs on illness often differed from biomedical ones and sometimes lay beliefs incorporated medical knowledge. Almost all participants had either used traditional herbs or consulted with a traditional healer following advice from other lay people, and they often referred to the advice from these alternative practitioners signifying how dominant this paradigm on what causes illness is, in this population. Traditional medicines and herbs are used by many Ugandans as part of their primary care because they are readily available and grown in many people’s homes and sometimes they are cheaper to use because often they do not need one to go for consultation but instead people access free advice from the lay network.[35]

The influence of lay beliefs on health behaviour was dominant probably because it was sometimes from people’s experiences and experiential accounts from peers may have sounded more convincing than information from health professionals. Also lay beliefs may have sounded more intelligible to the participants because they were the pervading ideas in the public arena. As Sabuni explains, people are socialised into these cultural ideas of health and illness since childhood [36] and therefore it is easier to comprehend these ideas than the biomedical ones. Also the Ugandan culture is mainly an oral culture (given low literacy levels) and reading or research on health issues is not common. Most people draw most of their information from media with traditional healers and alternative practitioners being aggressive in the use of media, especially the most commonly accessed form; radio. Their information may appeal to people more because it is more concordant with what they know [37] and probably relates to their discomfort directly. Additionally traditional healers tend to emphasise an explanation of the illness (as described by Kleinman et al above) as opposed to the disease. This appears to contribute to why people increasingly choose to go to alternative practitioners.[28] This has important implications for the training of health professionals in the approach to care of these participants and how to communicate with them.

Faith, cultural and religious beliefs were also important in influencing participants’ choices as observed in a patient who stopped medication because he believed that it was a way to show faith and if he had faith he would be cured. Heart failure was also conceptualised as due to spiritual causes, in line with another study done in Kenya. [38] This has important implications for public health education of faith, cultural and religious leaders who are important sources of information to the lay public.

### Reflexivity

The above research findings could have been influenced by the researcher who is a medical doctor. Her previous training and her role as a doctor could have influenced the way questions were asked and how data was analysed and interpreted. The researcher and the research team acknowledged this before data collection began and the researcher wrote down her assumptions before she generated the data to aid her reflect on how her assumptions influenced the data generated. Additionally, the data generated could have been influenced participants’ perceptions of the researcher. The researcher was seen by the participants as affiliated with the health care system having being introduced to them by the ward health professionals. We recognise the potential for this to have inhibited candid discussion on issues participants may have considered as sensitive for the health providers and if they thought that discussion of some issues would affect their care as demonstrated in the case when the participant did not want to disclose that he was using herbs. On recognition that participants might not be free to talk about herbs, a prompt eliciting data on use of herbs was included in the interview guide and the researcher was careful not to condemn the practice but ask about it as a usual practice in the community. The longitudinal nature of the interviews helped counteract this problem as relationships between the researcher and participants developed, especially in the subsequent interviews which appeared to have facilitated sharing of more sensitive information with time.

The researcher and research team at the beginning of the study anticipated role conflict issues and a plan was drawn on how to address them. Those issues that emerged during the data collection without prior anticipation were discussed with the research team and solutions were found. For example ethical issues arose during the interviews when a participant said he had taken wrong medications in the past believing that they were the ones that were effective. It is during these times that the researcher’s role as a researcher conflicted with her role as a doctor. As a researcher there was need to keep within the boundaries of a researcher to ensure rigorous methods by avoiding giving any intervention that would interfere with the research findings. However, as a doctor who has been socialized into the ethics and values of the profession the researcher had the moral obligation to keep the welfare of the participants and medical ethics in mind by ensuring beneficence and by not using people as means to an end. The issues were discussed with the research team and a decision was made to inform the ward team so that they could educate the patient on this. The researcher had debriefing sessions after the interviews with a member of the team with counselling skills. These were held at the end of every week during the data collection period.

The limitations of this study include the relatively narrow geographical field in which the study was conducted, also the research was only across two (albeit two of the dominant languages) of the numerous languages spoken in Uganda and therefore these findings may be limited to a particular sub-culture within Uganda.

## Conclusions

There is a high level of health illiteracy about HF in Uganda. Patients’ health decisions are largely influenced first by their local and cultural beliefs and then, for those who live longer, incorporate medical views. Medical knowledge is largely misconstrued by participants. Therefore there is an urgent need for health education and culturally appropriate information for patients and the wider society. More research into how best health providers can communicate and provide information that is accessible to participants is needed.

## Supporting information

S1 Appendix Interview guide. (ODT)

S2 Appendix Timing of face-to-face and telephone interviews and patient deaths. (ODT)
