# Supplementary material for: ‘I think my body has become addicted to those tablets’. Chronic heart failure patients’ understanding of and beliefs about their illness and its treatment: A qualitative longitudinal study from Uganda
Source: PLoS One. 2017 Sep 28;12(9):e0182876. doi: 10.1371/journal.pone.0182876 (PMC5619713; doi:10.1371/journal.pone.0182876)
Supplement: Supplementary file 1 [file pone.0182876.s001.odt]

Thank you for accepting to participate in this study. I am here to listen to your life story since your illness started. I am not looking for any particular answers please feel free to say anything that you feel is important to you and please take your time. With your permission I shall come back after sometime to hear more about your illness story.

 

- Tell me about your life  (socio-demographics)

Prompts  

-  Age, Occupation, family, friends, address.

- Tell me how your illness started (probably physical issues)

Prompts 

- How did you notice there was something wrong? What events lead to this?

- How bad was it? 

- When did you first notice that it was serious and how? 

- What did it mean to you?

- How did you feel?

- What did you do? And why? Any one influenced your actions? How did they do it?

- How have the above problems changed over time?

- What happened next? (diagnosis story)

Prompts 

- Tell me about the process by which your condition was determined?

- What do you know about your condition? How has this knowledge changed with time?

- How has this knowledge changed your life if at all?  

- Tell me about your social life? 

Prompts 

- How has life changed if at all and why? When did the changes occur ? What lead to the changes?

- How do you manage to live with the illness? How are you able to meet your day to day needs? Who has been most helpful to you during this time? And how?

- Tell me about your relationship with other people? Family, friends , neighbours. Has this changed? If at all why?

- How would you describe the kind of person you were before the illness and now? What contributed to this change?

- Could you describe a typical  day for you? Before the illness and after

- How if at all, have your thoughts and feelings about yourself changed since it all started? (psychological)

Prompts 

- What positive changes have occurred in your life since then?

- What negative changes if, any have occurred in your life since then?

- As you look back, are there any other events that stand out in your mind? Could you describe each one? How did this event affect how what happened? How did you respond to it?

- Tell me about the most important lessons you have learned through this illness?

- What helps you manage or go on? (coping and spiritual )

Prompts 

- What gives meaning to your life in this condition? Has this changed compared to past and how?

- What is your source of strength, hope, peace? What strengths you have discovered? 

- What do you most value about yourself now? What do others value in you?

- . How do you explain your condition?  Tell me what you understand of your illness.

Prompts 

- Where have you got information on your illness? Who gave it?

- How much information would you have liked to get? How much have you got so far? Why didn’t you get what you needed?

- Which information is important to you and why?

- How would you like this information to be given?

- Tell me about your care and treatment?

Prompts 

- What is it like to make routine visits for medical care?

- How do you access treatment? What process do you have to go through to get treatment?

- What is your opinion on the treatment used? How does it help if at all? What do you expect from the treatment? what do you think I the role of the medicines given to you?

- Do you have any concerns about the treatment? What is it like to have to take drugs every day?   How do you manage the medications? 

- How do you care for yourself? Why? What helps with this? What hinders this?

- What is your relationship with the health professionals? How do they appear to you?

- What do you expect from a hospital visit/ admission? What do you expect from care?  What would you like care for heart failure patients to be like?

- What leads to your change in condition to necessitate admission?

- After these experiences what advice would you give to someone who has just discovered they have this condition?

Prompts 

- Is there anything else you think I should know?

- Is there anything you would like to ask me?

Thank you for your time.
