# Supplementary material for: ‘I think my body has become addicted to those tablets’. Chronic heart failure patients’ understanding of and beliefs about their illness and its treatment: A qualitative longitudinal study from Uganda
Source: PLoS One. 2017 Sep 28;12(9):e0182876. doi: 10.1371/journal.pone.0182876 (PMC5619713; doi:10.1371/journal.pone.0182876)
Supplement: Supplementary file 2 [file pone.0182876.s002.odt]

| Patient 21 | dreamstime_l_3291533[1] | dreamstime_l_3291533[1]2NDchange in health | rip[1] |  |  |  |  |
| --- | --- | --- | --- | --- | --- | --- | --- |
| Patient 20 | dreamstime_l_3291533[1] | Mobile-Phone-Icon-300x300[1] | dreamstime_l_3291533[1]2ND scheduled | Mobile-Phone-Icon-300x300[1] | dreamstime_l_3291533[1]3RD Change in health |  |  |
| Patient 19 | dreamstime_l_3291533[1] | rip[1] |  |  |  |  |  |
| Patient 18 | https://sp.yimg.com/ib/th?id=HN.608015254774483494&pid=15.1&H=151&W=160&P=0 | Mobile-Phone-Icon-300x300[1] | dreamstime_l_3291533[1]2ND Change in health | rip[1] |  |  |  |
| Patient 17 | dreamstime_l_3291533[1] | large-No-Cell-Phones-Allowed-0-13970[1] | large-No-Cell-Phones-Allowed-0-13970[1] | large-No-Cell-Phones-Allowed-0-13970[1] | large-No-Cell-Phones-Allowed-0-13970[1] | large-No-Cell-Phones-Allowed-0-13970[1] | large-No-Cell-Phones-Allowed-0-13970[1] |
| Patient 16 | dreamstime_l_3291533[1] | rip[1] |  |  |  |  |  |
| Patient 15 | dreamstime_l_3291533[1] | large-No-Cell-Phones-Allowed-0-13970[1] | large-No-Cell-Phones-Allowed-0-13970[1] | large-No-Cell-Phones-Allowed-0-13970[1] | large-No-Cell-Phones-Allowed-0-13970[1] | large-No-Cell-Phones-Allowed-0-13970[1] | large-No-Cell-Phones-Allowed-0-13970[1] |
| Patient 14 | dreamstime_l_3291533[1] | large-No-Cell-Phones-Allowed-0-13970[1] | rip[1] |  |  |  |  |
| Patient 13 | dreamstime_l_3291533[1] | large-No-Cell-Phones-Allowed-0-13970[1] | Mobile-Phone-Icon-300x300[1] | dreamstime_l_3291533[1]2ND scheduled | Mobile-Phone-Icon-300x300[1] | Mobile-Phone-Icon-300x300[1] | dreamstime_l_3291533[1]3RD scheduled |
| Patient 12 | dreamstime_l_3291533[1] | dreamstime_l_3291533[1]2ND Change in health | rip[1] |  |  |  |  |
| Patient 11 | dreamstime_l_3291533[1] | large-No-Cell-Phones-Allowed-0-13970[1] | large-No-Cell-Phones-Allowed-0-13970[1] | large-No-Cell-Phones-Allowed-0-13970[1] | Mobile-Phone-Icon-300x300[1] | large-No-Cell-Phones-Allowed-0-13970[1] | large-No-Cell-Phones-Allowed-0-13970[1] |
| Patient 10 | dreamstime_l_3291533[1] | large-No-Cell-Phones-Allowed-0-13970[1] | large-No-Cell-Phones-Allowed-0-13970[1] | large-No-Cell-Phones-Allowed-0-13970[1] | large-No-Cell-Phones-Allowed-0-13970[1] | large-No-Cell-Phones-Allowed-0-13970[1] | large-No-Cell-Phones-Allowed-0-13970[1] |
| Patient 9 | dreamstime_l_3291533[1] | Mobile-Phone-Icon-300x300[1] | dreamstime_l_3291533[1]2ND scheduled | Mobile-Phone-Icon-300x300[1] | rip[1] |  |  |
| Patient 8 | dreamstime_l_3291533[1] | dreamstime_l_3291533[1]2ND Change in health | rip[1] |  |  |  |  |
| Patient 7 | https://sp.yimg.com/ib/th?id=HN.608015254774483494&pid=15.1&H=151&W=160&P=0 | Mobile-Phone-Icon-300x300[1] | Mobile-Phone-Icon-300x300[1] | dreamstime_l_3291533[1]2ND scheduled | Mobile-Phone-Icon-300x300[1] | Mobile-Phone-Icon-300x300[1] | dreamstime_l_3291533[1]3RD scheduled |
| Patient 6 | dreamstime_l_3291533[1] | Mobile-Phone-Icon-300x300[1] | dreamstime_l_3291533[1]2ND scheduled | Mobile-Phone-Icon-300x300[1] | Mobile-Phone-Icon-300x300[1] | dreamstime_l_3291533[1]3RD Change in health |  |
| Patient 5 | dreamstime_l_3291533[1] | Mobile-Phone-Icon-300x300[1] | Mobile-Phone-Icon-300x300[1] | dreamstime_l_3291533[1]2ND scheduled | Mobile-Phone-Icon-300x300[1] | Mobile-Phone-Icon-300x300[1] | dreamstime_l_3291533[1]3RD scheduled |
| Patient 4 | dreamstime_l_3291533[1] | Mobile-Phone-Icon-300x300[1] | Mobile-Phone-Icon-300x300[1] | dreamstime_l_3291533[1]2ND scheduled | Mobile-Phone-Icon-300x300[1] | Mobile-Phone-Icon-300x300[1] | dreamstime_l_3291533[1]3RD scheduled |
| Patient 3 | https://sp.yimg.com/ib/th?id=HN.608015254774483494&pid=15.1&H=151&W=160&P=0 | Mobile-Phone-Icon-300x300[1] | dreamstime_l_3291533[1]2ND | rip[1] |  |  |  |
| Patient 2 | dreamstime_l_3291533[1] | rip[1] |  |  |  |  |  |
| Patient 1 | dreamstime_l_3291533[1] | Mobile-Phone-Icon-300x300[1] | Mobile-Phone-Icon-300x300[1] | https://sp.yimg.com/ib/th?id=HN.608015254774483494&pid=15.1&H=151&W=160&P=0 2ND scheduled | Mobile-Phone-Icon-300x300[1] | rip[1] |  |
|  | 1ST Interview | Month 1 | Month2 | Month 3 | Month 4 | Month 5 | Month 6 |

KEY

Face to face interview with patient and their carer

Phone call 

C:\Users\v1enamuk_local\AppData\Local\Microsoft\Windows\Temporary Internet Files\Content.IE5\AQ01ZYGM\Mobile-Phone-Icon-300x300[1].png	      https://sp.yimg.com/ib/th?id=HN.608015254774483494&pid=15.1&H=151&W=160&P=0

Phone off

Died 

C:\Users\v1enamuk_local\AppData\Local\Microsoft\Windows\Temporary Internet Files\Content.IE5\0KJA0OUE\large-No-Cell-Phones-Allowed-0-13970[1].gif                                                                 C:\Users\v1enamuk_local\AppData\Local\Microsoft\Windows\Temporary Internet Files\Content.IE5\K4ZR5F76\rip[1].jpg   

                                                         

C:\Users\v1enamuk_local\AppData\Local\Microsoft\Windows\Temporary Internet Files\Content.IE5\O1MGYTRS\dreamstime_l_3291533[1].jpgFace to face interview with patient
